# Nanoscale Elastoplastic Wrinkling of Ultrathin Molecular Films

**DOI:** 10.3390/ijms222111732

**Published:** 2021-10-29

**Authors:** Gianfranco Cordella, Antonio Tripodo, Francesco Puosi, Dario Pisignano, Dino Leporini

**Affiliations:** 1 Dipartimento di Fisica “Enrico Fermi”, Università di Pisa, Largo B.Pontecorvo 3, I-56127 Pisa, Italy; gianfrancocordella@gmail.com (G.C.); antonio.tripodo@df.unipi.it (A.T.); francesco.puosi@pi.infn.it (F.P.); dario.pisignano@unipi.it (D.P.); 2 Istituto Nazionale di Fisica Nucleare (INFN), Sezione di Pisa, Largo B.Pontecorvo 3, I-56127 Pisa, Italy; 3 NEST, Istituto Nanoscienze-CNR, Piazza S. Silvestro 12, I-56127 Pisa, Italy; 4 Istituto per i Processi Chimico-Fisici-Consiglio Nazionale delle Ricerche (IPCF-CNR), Via G. Moruzzi 1, I-56124 Pisa, Italy

**Keywords:** ultrathin molecular films, nanoscale surface instabilities, elastoplasticity, wrinkling, molecular dynamics

## Abstract

Ultrathin molecular films deposited on a substrate are ubiquitously used in electronics, photonics, and additive manufacturing methods. The nanoscale surface instability of these systems under uniaxial compression is investigated here by molecular dynamics simulations. We focus on deviations from the homogeneous macroscopic behavior due to the discrete, disordered nature of the deformed system, which might have critical importance for applications. The instability, which develops in the elastoplastic regime above a finite critical strain, leads to the growth of unidimensional wrinkling up to strains as large as 0.5. We highlight both the dominant wavelength and the amplitude of the wavy structure. The wavelength is found to scale geometrically with the film length, λ∝L, up to a compressive strain of ε≃0.4 at least, depending on the film length. The onset and growth of the wrinkling under *small* compression are quite well described by an extended version of the familiar square-root law in the strain ε observed in macroscopic systems. Under *large* compression (ε≳0.25), we find that the wrinkling amplitude increases while leaving the cross section nearly constant, offering a novel interpretation of the instability with a large amplitude. The contour length of the film topography is not constant under compression, which is in disagreement with the simple accordion model. These findings might be highly relevant for the design of novel and effective wrinkling and buckling patterns and architectures in flexible platforms for electronics and photonics.

## 1. Introduction

Perfectly elastic rods buckle when subjected to an axial load exceeding a critical value, as first noted by Euler in 1744 [1,2]. The Euler buckling is an example of saddle-point elastic instability where disturbances drive the undeformed rod to a new stable buckled conformation [3]. The bifurcation corresponds to the disappearance of the restoring force opposing the lateral deformation of the rod, and the critical load can be straightforwardly found by linear elastic response theory [1,2,3]. Post-buckling effects, e.g., the magnitude of the lateral deflection, are instead inherently non-linear [4]. This leads to considerable theoretical difficulties in order to reach a comprehensive interpretation of the associated physical mechanisms which, moreover, are affected by deviations from ideality, e.g., imperfection sensitivity [5,6,7,8] and subsequent low reproducibility [9], plasticity [6,10,11,12,13] and spatial heterogeneity [14,15]. For an ideal elastic rod with hinged ends, the deflection curve obtained by the smallest, critical, uniaxial compression is described by one half-sine wave [1,2]. With an elastic foundation supporting the rod, i.e., an actual or effective continuous elastic medium with sufficient rigidity opposing the deflection of the rod, the resulting antagonistic effect penalizes long wavelengths and the Euler single buckle is replaced by a repeated pattern of multiple buckles [16,17]. These wavy patterns are ubiquitous [16] and have been observed over a huge range of multiple length scales from nanometers up to geological ones [18]. Moreover, they exhibit a rich variety of geometries, both extended wrinkles [19,20,21] and localized folds [22].

Over the last few decades, wrinkling—a possibly problematic aspect leading to material failure [23,24,25]—turned into an opportunity for a wide range of applications in 2D materials such as graphene [26], micro- and nanofluidics [27,28,29], adaptive materials [30], flexible electronics [31], film metrology [19,32], nanoelectromechanical systems (NEMS) [33], and for pattern formation in micro- and nanofabrication [22,34,35,36,37,38].

Our main goal in the present paper is to make progress on the characterization of surface instabilities when they occur on *nanometric* length scales. Nanometric instabilities are expected to be affected by non-ideality owing to the perceptible discrete nature of the system, requiring a microscopic model for accurate interpretation. As a first attempt, we address this aspect through Molecular-Dynamics (MD) simulations of the in-plane axial compression of an ultrathin molecular glassy film with nanometric height and length supported by a flat foundation. We were inspired by a few previous MD studies investigating microscopic wrinkling on layered copolymer materials [39] and substrate-supported graphene sheets [40]. Our study reveals that wrinkling occurs in the presence of *plasticity*. It is worth noting that, while huge efforts have been generally made in examining elastic systems, much less attention has been paid to consider the buckling and post-buckling behavior of plastic systems undergoing irreversible deformation even at macroscopic length scales [6,10,11,12,13]. We especially aim to characterize the wavelength λ and the amplitude *A* of the observed wrinkled structures due to their relevance in the applications mentioned previously. In the small deflection limit, mechanical models developed for *continuous, elastic* media lead to the following predictions [16,19,41,42,43]:

(1) $$\begin{array}{ccc} \lambda & = & \lambda_{0} \end{array}$$

(2) $$\begin{array}{ccc} A & \propto & \sqrt{\varepsilon -\varepsilon_{c}}\;\varepsilon \geq \varepsilon_{c} \end{array}$$

where $\lambda_{0}$ is a constant set by the elastic properties of the structure under deformation, ε is the longitudinal strain $\varepsilon =\left(L_{0}-L\right)/L_{0}$ of the uniaxial compression reducing the longitudinal size from the original length $L_{0}$ to *L*, and $\varepsilon_{c}$ is a critical strain below which no wrinkling is observed. Equations (1) and (2) state that, if the deformation exceeds the critical strain, the wrinkling amplitude *changes* with *no* changes in the wavelength. Going beyond the small deformation limit, experiments show that Equation (1) is untenable since wrinkles exhibit shorter and shorter wavelengths upon increasing compression [20,43]. This is not solved by a non-linear analysis of wrinkles, which would still predict Equation (1) though extending the range of validity of Equation (2) [42]. For large deformations of stiff films adhered to a soft substrate, the accordion approximation is often invoked. According to this approximation, the contour length of the film is preserved after it buckles, e.g., see [16,20,22,43]. If the number of wrinkles does not change during compression, the accordion approximation predicts [20]: (i) the decrease in the wavelength according to the scaling λ∝L and (ii) the increase in the amplitude according to Equation (2) with $\varepsilon_{c}=0$ for small amplitudes:

(3) $$\begin{array}{ccc} \lambda & = & \lambda_{0}\left(1-\varepsilon \right) \end{array}$$

(4) $$\begin{array}{ccc} A & = & \frac{\lambda_{0}}{\pi }\sqrt{\varepsilon } \end{array}$$

In a different approach, considering the contour length constancy in the minimization of the total elastic energy leads to Equations (1) and (4) in the wrinkling regime [16,22] and allows for the description of the localized folding regime [22]. A finite-deformation buckling theory, based on the non-linear elastic neo- Hookean constitutive law, has also been developed to investigate the wrinkling of a thin stiff film on a compliant substrate up to ∼30–40% compression [31,43,44]. The approach recovers both Equations (2) and (3) for small deformations and extends them to large deformations, showing good agreement with experiments.

Our results suggest the partial robustness of some predictions provided by mechanical models developed for continuous media and propose a suitable generalization. More explicitly, we find:

- $\varepsilon_{c}>0$, namely, the onset of wrinkling occurs at *finite* compression.
- The wavelength dependence on the compression is accounted for by Equation (3) up to remarkably large deformations.
- Under *small* compression, the wrinkling amplitude *A* grows according to an extended version of Equation (2), accounting for some rounding of the bifurcation at $\varepsilon =\varepsilon_{c}$.
- Under *large* compression, the amplitude *A* grows according to the geometrical law that the wrinkling *cross section* is nearly constant.
- The contour length of the topography of the wrinkled film, *ℓ*, changes under compression, which is in disagreement with the simple accordion model.

Overall, this set of results might be highly relevant for the design of novel, effective and reproducible wrinkling and buckling patterns for flexible platforms in electronics and photonics.

## 2. Results and Discussion

### 2.1. Elastic and Plastic Regimes of the Thin Film

**Figure 1:**
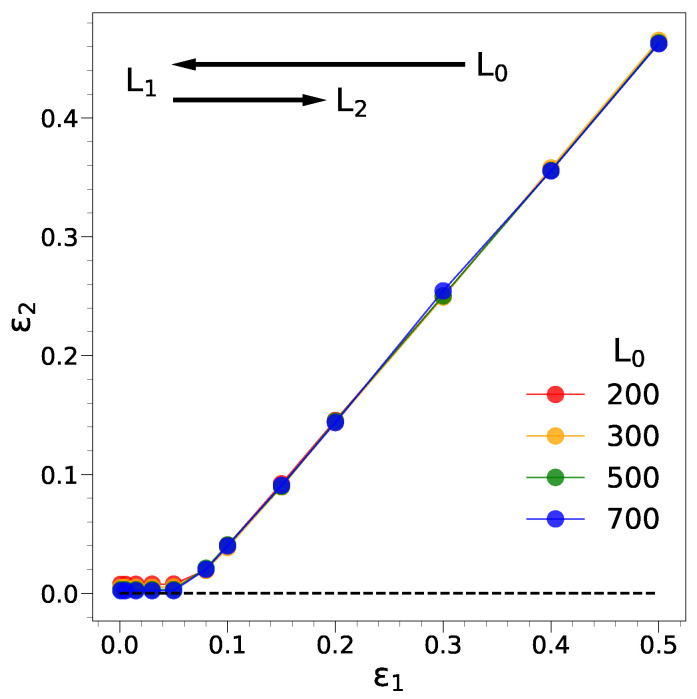
Mechanical response of the film under a compression/decompression cycle. The film with initial length $L_{0}$ is uniaxially compressed up to $L=L_{1}$ with strain $\varepsilon =\varepsilon_{1}$ and later decompressed. The decompression leaves the film with residual deformation $L=L_{2}$ and strain $\varepsilon =\varepsilon_{2}$. Elastic response, corresponding to $\varepsilon_{2}=0$, i.e., $L_{2}=L_{0}$, is observed up to about $\varepsilon_{1}≃0.05$. Plastic effects are apparent at larger compressions and virtually independent of the film length. The film lengths are in units of the reference length σ, the approximate monomer diameter. In the case of polyethylene (polystyrene), one deduces a reference length σ=5.3(9.7) Å; see Section 3.

To characterize the mechanical response, we perform a cycle where the film, initially with length $L_{0}$, is uniaxially compressed up to $L_{1}$, $L_{1}<L_{0}$, with the corresponding strain $\varepsilon =\varepsilon_{1}$. Later, the film is decompressed. The decompression leaves the sample with length $L_{2}$, $L_{2}\leq L_{0}$ and strain $\varepsilon =\varepsilon_{2}$. With an elastic response, $\varepsilon_{2}=0$ and $L_{2}=L_{0}$, whereas plasticity is signalled by $\varepsilon_{2}>0$ and $L_{2}<L_{0}$. Figure 1 provides information about $\varepsilon_{2}$ vs. $\varepsilon_{1}$. It can be seen that for $\varepsilon_{1}≲0.05$, no residual deformation is left. Plastic effects are apparent at larger compressions in a way which is virtually independent of the film length.

### 2.2. Buckling Wavelength

**Figure 2:**
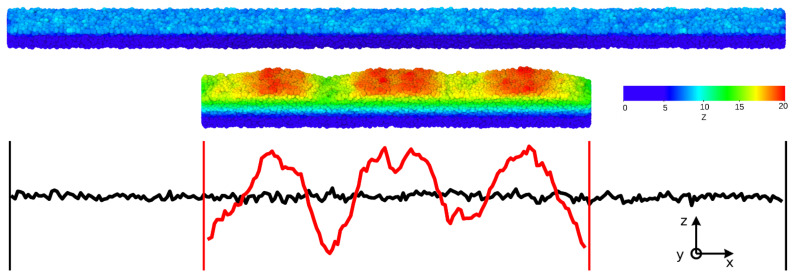
Slices with depth 13σ of a glassy film of pristine height $h_{0}=9\sigma$ and length $L_{0}$ ($L_{0}=300\sigma$) compressed to length $L=L_{0}/2$. Hotter colors indicate particles with higher elevations *z*. The black and red curves represent the surface topography of the uncompressed and compressed systems, respectively. Both the elevation and the film lengths are in units of the reference length σ, the approximate monomer diameter. In the case of polyethylene (polystyrene), one deduces a reference length σ=5.3(9.7) Å; see Section 3.

Figure 2 shows a representative sketch of the wavy pattern developing on the top, free surface of the film when the system undergoes uniaxial compression (visualization performed by OVITO—the Open Visualization Tool [45]). Wrinkles are observed along the $\hat{x}$ direction with substantial one-dimensional character, i.e., with features that are largely $\hat{y}$-independent.

**Figure 3:**
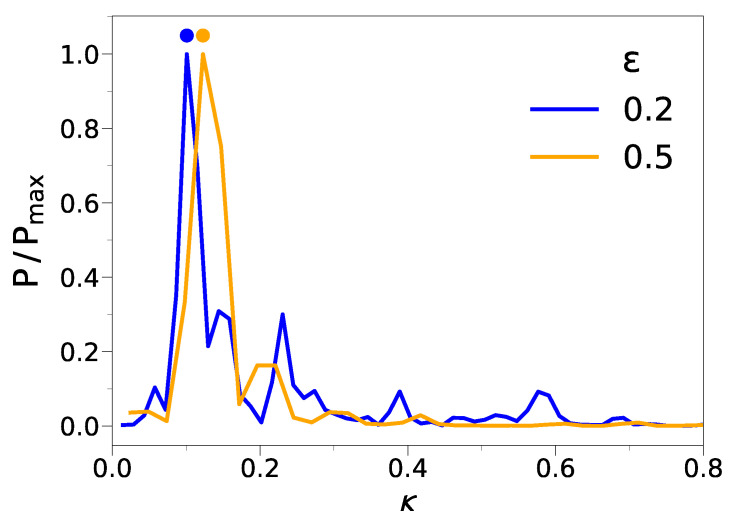
Normalized power spectrum of the FT of the topography of the thin film shown in Figure 2 at two different compressions. The plot gives evidence that wrinkling is characterized by a well-defined dominant wavenumber $\kappa_{max}$ (marked by a dot). The wavenumbers are in units of $\sigma^{-1}$; see Section 3.

Figure 3 shows the Fourier transform (FT) of the topography of the film in Figure 2 at two different compressions. A dominant wavenumber $\kappa_{max}$ clearly emerges. Due to the disordered and discrete nature of the molecular film, the value of $\kappa_{max}$ depends on the particular sample under examination. Therefore, to capture significant information about the dominant wavenumber, we averaged the wavenumber associated with the FT maximum, $\kappa_{max}$, over all the replicas of the film. Henceforth, the resulting average will be denoted as *k*.

**Figure 4:**
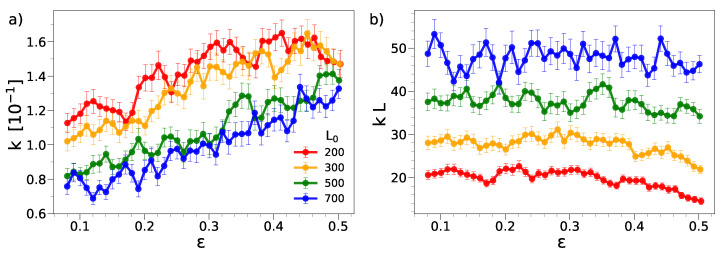
Dependence of the wavenumber (**a**) and reduced wavenumber (**b**) upon compression. The wavenumbers are in units of $\sigma^{-1}$; see Section 3.

Figure 4a shows the dependence of the wrinkling wavenumber on the deformation strain for different film lengths. The plot covers a range with appreciable wrinkling amplitude, roughly ε≳0.08. According to Figure 1, plastic effects are not negligible in this range. For the longer films, the wavenumber exhibits a steady increase when the compression is increased, i.e., the wavelength *decreases*. The decrease in the wavelength is also observed in the shortest films up to significant compressions, ε≃0.4, levelling off at larger compressions. Noticeably, looking at the behavior for $L_{0}\geq 500$, it appears that we are approaching a limit in which, for a given compression, the wavenumber is unaffected by the film length, pointing to the intrinsic nature of the phenomenon, namely that the specific wavelength, at a given deformation, is controlled only by the system’s microscopic details.

Figure 4b inspects the product kL. It is seen that the product kL is nearly constant in the wide range of 0.08≲ε≲0.4 (for the longest film there is a tendency to extend the upper boundary up to about 0.5). This finding is consistent with the hypothesis that the dominant wavelength is proportional to the length of the compressed sample, λ∝L, which is equivalent to Equation (3).

Our results show that the geometric law k∝1/L holds only if the film is long enough. This could be due to the presence of the two walls at the ends of the film at X=0,L. We suggest that each wall constrains the adjacent region of the film with width $\xi_{x}$ along the $\hat{x}$ direction and $\xi_{x}$ being poorly dependent on the film length *L* if $\xi_{x}≲L$, and it could be argued that k∝1/L only in films with length $≳2\xi_{x}$. To provide a rough estimate of $\xi_{x}$, we note that, upon compressing the shortest film with $L_{0}=200$, the quantity kL starts to decrease around ε∼0.5, corresponding to the length L=100. This suggests that $\xi_{x}∼100/2=50$. The above picture needs confirmation by additional studies which are beyond the scope of the present paper and postponed to later investigations.

### 2.3. Buckling Amplitude

**Figure 5:**
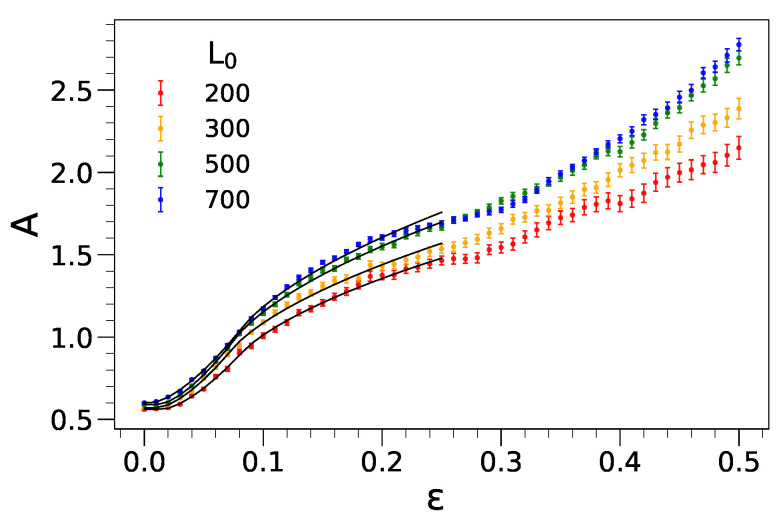
Wrinkling amplitude over the whole range of uniaxial compression. The superimposed lines are the best-fit curves according to Equation (5). The best-fit parameters are summarized in Table 1. The amplitude is in units of σ, see Section 3.

We evaluate the average amplitude of the wrinkling *A* over all the film replicas with initial length $L_{0}$ when compression increases. The results are shown in Figure 5. The overall pattern shows three characteristic regions. For 0≲ε≲0.02, one has A≃0.6. In this regime, our PH algorithm, see Section 3, simply reveals the intrinsic roughness of the film, in the order of the particle radius, and no wrinkling is detected. In the range 0.02≲ε≲0.25, the amplitude *A* increases with the compression and reaches an inflection point at ε∼0.25 (the position of the inflection point is anticipated to be model-dependent). Finally, in the range 0.25≲ε≤0.5 a new steeper growth regime of the wrinkling amplitude sets in by increasing the compression. The small and large compression regimes are analyzed in depth in the following.

#### 2.3.1. Small Compression: ε≲0.25

We interpret the amplitude increase in the range 0≲ε≲0.25 as the nanometric counterpart of the square-law amplitude following the bifurcation at $\varepsilon_{c}$ observed at macroscopic length scales, i.e., Equation (2). To account for the rounding of the wrinkling onset, ascribed to the distribution of the microscopic arrangements of each replica of the film with given length, we assume that the critical onset $\varepsilon_{c}$ exhibits a rectangular distribution with lower and upper bounds $\varepsilon_{m}$ and $\varepsilon_{m}+\Delta$, respectively. This yields the following fit function:

(5) $$A\left(\varepsilon \right)=A_{0}+\frac{2b}{3\Delta }\left[{\left(\varepsilon -\varepsilon_{m}\right)}^{3/2}\Theta \left(\varepsilon -\varepsilon_{m}\right)-{\left(\varepsilon -\varepsilon_{m}-\Delta \right)}^{3/2}\Theta \left(\varepsilon -\varepsilon_{m}-\Delta \right)\right]$$

where Θ(x) is the Heaviside step function (Θ(x)=0 for x<0 and Θ(x)=1 otherwise). $A_{0}$ and *b* are the residual amplitude and a scale parameter, respectively. Since we take the former from the pre-wrinkling regime, the fit function, Equation (5), has three adjustable parameters, *b*, $\varepsilon_{m}$ and Δ. The best-fit parameters are summarized in Table 1 and the resulting best-fit curves are drawn up to ε=0.25 in Figure 5.

#### 2.3.2. Large Compression: 0.25≲ε≤0.5

Obviously, Equation (5) is unable to account for the change in concavity which is observed at the inflection point ε∼0.25 in Figure 5. The same conclusion is reached by considering the accordion approximation, predicting Equation (4). The accordion approximation is based on the assumption that the contour length of the wrinkling, *ℓ*, is unaffected by the film compression. We have inspected this assumption and evaluated the contour length of the topography of a film of original length $L_{0}$ under compressive strain ε, $\ell (\varepsilon ,L_{0})$. Our analysis is rather preliminary. Nonetheless, it points to the conclusion that the contour length is *not* constant and hints at the scaling $\ell (\varepsilon ,L_{0})\propto L$. In fact, one finds ℓ(0,200)=201 and ℓ(0.5,200)=104; ℓ(0,500)=502 and ℓ(0.5,500)=259; ℓ(0,700)=704 and ℓ(0.5,700)=363. A simple explanation of the scaling comes from the remark that the number of the wrinkles of our thin film is constant during compression and their amplitude is small with respect to their wavelengths. Then, $\ell \propto \sqrt{A^{2}+{\left(2\pi /k\right)}^{2}}≃2\pi /k\propto L$, where the last passage follows from Figure 4b, i.e., k∝1/L.

**Figure 6:**
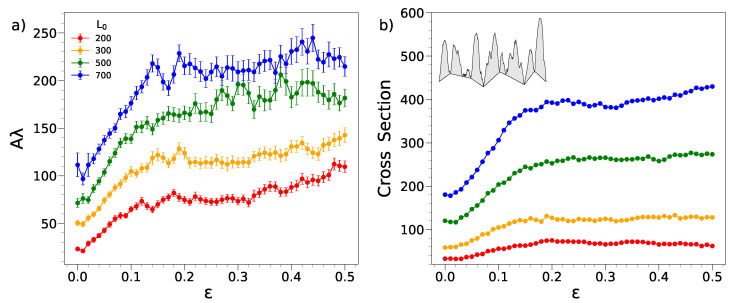
(**a**) Product of the average amplitude *A* and wavelength λ of the wrinkling. The product is a proxy of the cross section of a wrinkle. The relative increases in the amplitude in the range 0.25≤ε≤0.5 with respect to the overall increase are 0.43,0.32,0.15,0.05, from the shortest to the longest film. The curves corresponding to $L_{0}=300,500,700$ have been shifted upwards by 20,40,60 units, respectively, for clarity. (**b**) Overall cross section of the wrinkling (error bars smaller than the dot size). The sketch illustrates the evaluation according to the PH algorithm detailed in Section 3. The relative increases in the amplitude in the range 0.25≤ε≤0.5 with respect to the overall increase are −0.33,0.05,0.09,0.14 from the shortest to the longest film. Both the quantity Aλ and the cross section are in units of $\sigma^{2}$; see Section 3.

We find that an explanation of the amplitude growth in the hig-compression regime comes from consideration of the *cross section* of the wrinkles. To this aim, we first consider a proxy of this quantity, namely the product of the amplitude Aλ where λ is the average of the quantity $2\pi /\kappa_{max}$ over the replicas of a given film. To test the robustness of the procedure, we also evaluated the product of the amplitude of a given film with $2\pi /\kappa_{max}$ and then averaged the replicas. The results are indistinguishable from the quantity Aλ. Figure 6a plots the product Aλ while increasing the compression. It is clearly seen that, after an initial steep growth, the product exhibits a mild increase, especially for the longest films in the wide deformation range 0.25≲ε≤0.5. To gain further insight, we evaluated the overall cross section of *all* the wrinkles according to the procedure detailed in Section 3. The results are presented in Figure 6b. It is seen that the more precise evaluation exposes in a neater way that above ε≃0.25 the cross section of the wrinkles changes *very weakly* apart from the shorter film where the influence of the compressing walls is anticipated to be larger. Since the wrinkling pattern is nearly *y*-independent, the finding that the overall cross section of the wrinkles does not change appreciably after a first increase suggests that the volume of the wrinkling pattern does the same.

In other words, our results indicate that the net flux of matter exchanged between the wrinkling pattern and the underlying bulk region of the film is quite small for ε≳0.25. It is a simple matter to show that the near constancy of the cross section recovers the upward concavity of the curve *A* vs ε at large compression. In fact, A∝1/λ∝1/(1−ε), where the last passage follows from Equation (3).

## 3. Model and Methods

MD simulations are carried out with the open-source software LAMMPS [46,47]. We consider a dense ensemble of chains made of linear trimers. Bending and torsional interactions are neglected, i.e., the chain is completely flexible. Adjacent monomers in the same chain are bonded by an harmonic potential $k{\left(\ell -\ell_{0}\right)}^{2}$/2. Pairs of non-adjacent monomers of the same chain, as well as monomers belonging to different chains, interact through the truncated Lennard–Jones (LJ) potential:

(6) $$\begin{array}{ccc} U_{LJ}\left(r\right) & = & 4\varepsilon \left[{\left(\frac{\sigma }{r}\right)}^{12}-{\left(\frac{\sigma }{r}\right)}^{6}\right]\;+\;U_{cut}\;r\leq r_{cut} \end{array}$$

(7) $$\begin{array}{ccc} & = & 0\;otherwise \end{array}$$

where $r_{cut}=2.5\;\sigma$. The vertical shift $U_{cut}$ ensures that the potential is continuous at $r=r_{cut}$. Henceforth, both the monomer mass *m* and the Boltzmann constant $k_{B}$ have unit values, with energies expressed in units of ε, temperature in units of $\varepsilon /k_{B}$ and lengths in units of σ, roughly the monomer diameter. A suitable comparison between experimental and numerical data establishes correspondence between MD and reference units. For instance, in the case of polyethylene (polystyrene), one deduces a reference length σ=5.3(9.7) Å, a reference mass m=42.3(364) g/mol and a reference energy $\varepsilon /k_{B}=443\left(490\right)$ K [48].

We set the bond equilibrium length to $\ell_{0}=0.9\;\sigma$ and elastic constant to $k=1110\;\varepsilon /\sigma^{2}$ [49]. The present model is known to resist crystallization [49,50,51] and to capture the phenomenology of bulk and confined organic molecular systems [49].

The preparation of the solid amorphous film with the initial length $L_{0}$ and height $h_{0}=9\sigma$ was performed as follows. First, the system is placed in a box with length $L_{0}$ and fixed depth 13σ, along the $\hat{x}$ and $\hat{y}$ directions, respectively. Periodic boundary conditions are ensured across the *y* axis. The film is initially equilibrated in the NPT ensemble (constant particle number, constant temperature, constant pressure) at temperature T=1.5 under confinement by four smooth, i.e., structureless, rigid and flat walls. The four walls are located at X=0, $X=L_{0}$, Z=0 and $Z=h_{0}$. Monomers at distance $r_{⊥}$ from a wall experience a force perpendicular to the latter due to the potential:

(8) $$U_{wall}\left(r\right)=\varepsilon \left[\frac{2}{15}{\left(\frac{\sigma }{r_{⊥}}\right)}^{9}-{\left(\frac{\sigma }{r_{⊥}}\right)}^{3}\right]\;.$$

The equilibration is terminated when the normalized time correlation function of the end-to-end vector of a single chain is less than 0.1. Then, the system is instantaneously quenched at T=0.001 and the wall at $Z=h_{0}$ is removed to create a free, flat, upper interface, while leaving the vertical walls at X=0, $X=L_{0}$ and the lower horizontal surface at Z=0, the latter acting as a foundation; see Figure 2. This choice leaves only *one* free surface where wrinkling takes place upon uniaxial compression, the upper one of the film, thus simplifying considerably the interpretation of the results.

Finally, to ensure mechanical equilibration (negligible total force on each particle), a suitable energy minimization was carried out using a steepest descent algorithm. We studied films with lengths $L_{0}=200,300,500,700$. The total number of particles of a film depends on the length: N= 24,000 ($L_{0}=200$), N= 36,000 ($L_{0}=300$), N= 60,000 ($L_{0}=500$) and N= 84,000 ($L_{0}=700$), resulting in a number density ρ∼1 for all the films. For a given length $L_{0}$, 70 replicas are prepared to ensure significant statistics. The uniaxial in-plane compression of the film proceeds stepwise according to the Athermal Quasi-Static (AQS) procedure [52]. In each step, the coordinates of the particles constituting the film as well as the position of the vertical walls are affinely scaled along the $\hat{x}$ direction by the homogeneous strain $\delta \varepsilon =2.5\cdot 10^{-5}$ with a later energy minimization to recover the mechanical equilibrium. The step was iterated to reach the desired film length *L*, the distance between the vertical planes. To expand the film, if needed, an analogous procedure is followed.

Given the uniaxial nature of the deformation, the analysis of the wrinkling involves an average along the depth of the film, i.e., across the $\hat{y}$ direction, in addition to the average over all the replicas of the films. In more detail, to study the topography of the free surface along $\hat{z}$, first we created a grid in the foundation, i.e., the horizontal plane at z=0. The grid was formed by bins with size 1×2 along $\hat{x}$ and $\hat{y}$, respectively. The size ensures that three particles at least being located at the free surface have suitable (x,y) coordinates to be assigned to the same bin. Then, for each bin, an average height was evaluated considering the three particles at larger distances from the foundation. The procedure returned a two-dimensional scalar function which was finally averaged along $\hat{y}$ to provide a one-dimensional representation of the average topography of the free surface. Henceforth, for sake of simplicity, the latter quantity will be referred to as the topography. Three basic quantities were evaluated, namely the wavelength of the wrinkles, their amplitude and cross-section. The wavelength was evaluated by spatial discrete fast Fourier transform of the topography with rectangular windowing. The evaluation of the amplitude of the wrinkling was assisted by a persistent homology (PH) algorithm, an algebraic method which is robust against perturbations of input data and used in topological data analysis for measuring the features of shapes and functions that persist across multiple scales [53,54,55]. To this aim, the local extrema of the film topography were first identified. Then, to assign the physically acceptable contributions to the wrinkling amplitude, each local maximum was paired to its closest local minimum, according to their persistence *p*, a key concept elaborated on in the PH theory. All the pairs with $p<p_{0}$ were removed. Roughly speaking, $p_{0}$ sets the minimum physically acceptable amplitude value that is distinguishable from the inherent roughness of the film interface, i.e., monomers with diameter sizes in the order of ∼σ=1. Therefore, $p_{0}$ was adjusted in the range $0.5\leq p_{0}\leq 1$. Going into detail, we took $p_{0}=1$ for ε≥0.1 where wrinkles are apparent, whereas in the range 0≤ε<0.1, we adopted the linear interpolation $p_{0}\left(\varepsilon \right)=5\left(\varepsilon -0.1\right)+1$. The computation of the transverse cross section of the wrinkles was performed by joining the minima of the PH-filtered topography with a polygonal chain and calculating the area bounded by this baseline and the height profile.

## 4. Conclusions

We carried out an extensive numerical investigation of the nanoscale surface instability of supported glassy ultrathin films under uniaxial compression. To the best of our knowledge, this is the first ever attempt to investigate the role of the discrete, disordered nature of the deformed system. The instability, which develops in the elastoplastic regime above a critical strain $\varepsilon_{c}≃0.02$, leads to the growth of wrinkling up to ε=0.5. We investigated both the dominant wavelength λ and the amplitude *A* of the wavy structure. The wavelength scales with the film length, λ∝L, up to ε≃0.4 at least, depending on the film length. The amplitude exhibits a complex dependence on the compression with two distinct regimes, pertaining to small and large compressions, being clearly separated by an inflection point occurring at ε∼0.25. For *small* compressions, the initial growth is well described by an extended form of the familiar square-root law with respect to the strain ε observed in macroscopic homogeneous systems. At *large* compressions, we find that the cross section of the wrinkling is nearly constant, offering a novel interpretation of the large wrinkling amplitude. The contour length of the film topography changes under compression, which is at odds with the simple accordion model, and a simple scaling law is tentatively suggested.

## Figures and Tables

**Table 1:** Best-fit parameters of Equation (5).

$L_{0}/\sigma$	b/σ	$\varepsilon_{m}$	Δ
200	2.05 ± 0.01	0.016 ± 0.001	0.068 ± 0.003
300	2.20 ± 0.02	0.012 ± 0.001	0.063 ± 0.003
500	2.45 ± 0.01	0.012 ± 0.001	0.067 ± 0.002
700	2.56 ± 0.02	0.006 ± 0.002	0.078 ± 0.004

## Data Availability

The data presented in this study are available from the corresponding author upon request.

## Abbreviations

The following abbreviations are used in this manuscript:

AQS	Athermal Quasi-Static
FT	Fourier Transform
MD	Molecular Dynamics
PH	Persistent homology
